# Tobacco smoking, polymorphisms in carcinogen metabolism enzyme genes, and risk of localized and advanced prostate cancer: results from the California Collaborative Prostate Cancer Study

**DOI:** 10.1002/cam4.334

**Published:** 2014-10-30

**Authors:** Ahva Shahabi, Román Corral, Chelsea Catsburg, Amit D Joshi, Andre Kim, Juan Pablo Lewinger, Jocelyn Koo, Esther M John, Sue A Ingles, Mariana C Stern

**Affiliations:** 1 Department of Preventive Medicine, Keck School of Medicine of USC, Norris Comprehensive Cancer Center, University of Southern CaliforniaLos Angeles, California, 90033; 2 Department of Epidemiology, Harvard School of Public HealthBoston, Massachusetts, 02115; 3 Cancer Prevention Institute of CaliforniaFremont, California, 94538; 4 Division of Epidemiology, Department of Health Research and Policy and Stanford Cancer Institute, Stanford University School of MedicineStanford, California, 94305

**Keywords:** CYP1A2, prostate cancer, smoking

## Abstract

The relationship between tobacco smoking and prostate cancer (PCa) remains inconclusive. This study examined the association between tobacco smoking and PCa risk taking into account polymorphisms in carcinogen metabolism enzyme genes as possible effect modifiers (9 polymorphisms and 1 predicted phenotype from metabolism enzyme genes). The study included cases (*n* = 761 localized; *n* = 1199 advanced) and controls (*n* = 1139) from the multiethnic California Collaborative Case–Control Study of Prostate Cancer. Multivariable conditional logistic regression was performed to evaluate the association between tobacco smoking variables and risk of localized and advanced PCa risk. Being a former smoker, regardless of time of quit smoking, was associated with an increased risk of localized PCa (odds ratio [OR] = 1.3; 95% confidence interval [CI] = 1.0–1.6). Among non-Hispanic Whites, ever smoking was associated with an increased risk of localized PCa (OR = 1.5; 95% CI = 1.1–2.1), whereas current smoking was associated with risk of advanced PCa (OR = 1.4; 95% CI = 1.0–1.9). However, no associations were observed between smoking intensity, duration or pack-year variables, and advanced PCa. No statistically significant trends were seen among Hispanics or African-Americans. The relationship between smoking status and PCa risk was modified by the *CYP1A2* rs7662551 polymorphism (*P*-interaction = 0.008). In conclusion, tobacco smoking was associated with risk of PCa, primarily localized disease among non-Hispanic Whites. This association was modified by a genetic variant in *CYP1A2*, thus supporting a role for tobacco carcinogens in PCa risk.

## Introduction

Tobacco smoking is associated with an increased risk of several cancers, yet its carcinogenic role in the prostate is not clearly established [1, 2]. Whereas several epidemiologic studies do not support an association [2], a meta-analysis of 24 prospective cohort studies reported an estimated overall increased risk of PCa- and PCa-related mortality associated with tobacco smoking [1]. A 2009 review of the epidemiological literature further supported an association between tobacco smoking and aggressive PCa [3]. Possible factors contributing to inconsistencies in the literature include the heterogeneity in study designs, the varying smoking status definitions, the lack of details on smoking history and cessation, and lack of consideration of stage and tumor grade. Moreover, risk of PCa associated with tobacco smoking may also differ by race/ethnicity. Most studies examining the association between PCa risk and tobacco smoking predominantly include non-Hispanic White men, with only a few in African-Americans (AA) [4, 5] or Hispanics [6].

Tobacco smoke contains a wide variety of chemical carcinogens, including polycyclic aromatic hydrocarbons (PAHs), aromatic amines, heterocyclic amines (HCAs), and *N*-nitroso compounds (NOCs) [7]. The prostate gland is able to metabolize many of these chemicals into activated carcinogens [8–11], suggesting a plausible link between tobacco smoking and prostate carcinogenesis. In support of this, a prior study has reported associations between tobacco smoking and the presence of PAH-DNA adducts in the prostate, which varied by race and were modified by genetic variants involved in PAH metabolism [12]. To date, few studies have evaluated polymorphisms in tobacco carcinogen metabolism enzymes as possible modifiers of the association between tobacco consumption and PCa risk, in particular among different racial/ethnic populations [5, 13, 14].

In this study, we evaluated associations between tobacco smoking and risk of localized and advanced PCa using data from the California Collaborative Prostate Cancer Study, a large population-based case–control study in non-Hispanic White, AA, and Hispanic men. We also considered the role of polymorphisms in selected genes that code for tobacco carcinogens metabolism enzymes (*GSTP1*, *PTGS2, CYP1A2, CYP2E1, EPHX1, CYP1B1, UGT1A6*, *NAT2*, *GSTM1,* and *GSTT1*) as potential modifiers of the relationship between tobacco smoking and PCa risk.

## Materials and Methods

The California Collaborative Prostate Cancer Study was conducted in Los Angeles County (LAC) and in the San Francisco Bay area (SFBA) and used similar protocols and a common structured questionnaire administered in person. The characteristics of the study population and participation rates have been previously described [15, 16]. Briefly, newly diagnosed PCa cases were identified through the LAC and Greater Bay Area cancer registries. At both study sites, patients with intracapsular PCa were classified as localized cases and patients with extracapsular extension of the tumor, and/or extension into adjacent surrounding tissue, or regional lymph nodes, or metastasis to other areas of the body, were classified as advanced cases (Surveillance, Epidemiology, and End Result [SEER] 1995 clinical and pathologic extent of disease codes 41–85).

### Study population

#### San Francisco Bay Area

Eligible localized cases aged 40–79 years diagnosed from 1997 to 1998 were randomly sampled among non-Hispanic White men (15% sample) and AA men (60% sample). Eligible advanced cases aged 40–79 years included all non-Hispanic White men and all AA men diagnosed from 1997 to 2000. Controls were identified through random-digit dialing and, for men aged ≥65 years, through random selections from the rosters of beneficiaries of the Health Care Financing Administration, and they were frequency matched to advanced cases on race/ethnicity and the expected 5-year age distribution of cases. The in-person interview was completed by 208 localized cases (73 AA and 135 non-Hispanic Whites), 568 advanced cases (118 AA and 450 non-Hispanic Whites), and 545 controls (90 AA and 455 non-Hispanic Whites).

#### Los Angeles County

AA, Hispanic, and non-Hispanic White males diagnosed with PCa from 1999 to 2003 were identified by rapid case ascertainment through the LAC Cancer Surveillance Program. Controls were identified through a standard neighborhood walk algorithm [17] and were matched to cases on age (±5 years) and race/ethnicity. The in-person interview was completed by 1184 cases (351 AA, 333 Hispanics and 500 non-Hispanic Whites), including 631 with advanced PCa and 553 with localized PCa, and 594 controls (163 AA, 122 Hispanics and 309 non-Hispanic Whites).

Blood or mouthwash samples were collected for 1164 advanced cases, 553 localized cases (in LAC only), and 1119 controls. Written informed consent was obtained from all the study participants at the time of in-person interview. The study received approval from the institutional review boards at the Cancer Prevention Institute of California and the University of Southern California.

### Data collection

A structured questionnaire, administered at the participant's home, asked about demographic background, medical history, body size, tobacco use, and other lifestyle factors. The interviewers also measured height and weight. Usual dietary intake during the reference year (calendar year before diagnosis for cases or before selection into the study for controls) was assessed using a 74-item food frequency questionnaire (FFQ) that was adapted from the Block Health History and Habits Questionnaire [18]. An aggregate level socioeconomic status (SES) variable was derived from 2000 census data as previously described [19]. Body mass index (BMI) was calculated as self-reported weight (in kg) in the reference year and divided by height (in meters) squared measured at the time of the interview and categorized as normal weight (BMI < 25), overweight (BMI 25–29.9) and obese (BMI ≥ 30). Underweight men (BMI < 18.5, *n* = 15) were grouped with normal-weight men. For individuals with missing information on self-reported weight (1 case and 1 control), BMI was calculated using measured weight. For individuals who declined height measurement (4.9% of cases, 4.8% of controls), BMI was calculated using self-reported height.

### Tobacco consumption variables

The questionnaire assessed lifetime histories of smoking (cigarettes, cigars, pipe), tobacco chewing, and use of tobacco snuff. Information was collected on the ages at which men started and stopped tobacco consumption, and years and amount of tobacco consumption (cigarettes per day, cigars per week, pipes per week, chewing tobacco per week, cans of snuff per week). Ever tobacco smoking (not including tobacco chewing or snuff) was defined as smoking at least one cigarette a day and/or one cigar/pipe a week for 6 months or longer, and former smokers were defined as individuals who quit smoking prior to the reference year. The following variables were evaluated: history of tobacco smoking (ever/never), smoking status (never, former, current), age started to smoke (years), duration of smoking (years), type of tobacco used (cigarettes, cigars, pipes) and cigarettes smoked per day (lifetime average), and pack-years of cigarette smoking (ratio of the number of cigarettes smoked per day to 20 cigarettes, which is the current number of cigarettes per pack, multiplied by the total number of years smoked), and years passed since quitting smoking among former smokers. Variables were dichotomized based on the median value among controls who ever smoked tobacco.

### Polymorphisms data

As previously reported [20], genotype information was available for 11 single-nucleotide polymorphisms (SNPs) in eight genes reported to impact enzyme function: *GSTP1* Ile105Val (rs1695) [21], *PTGS2* -765 G/C (rs20417) [22], *CYP1A2* -154 A/C (rs762551) [23], *CYP2E1* -1054C>T (rs2031920), *EPHX1* Tyr113His (rs1051740) [24], *CYP1B1* Leu432Val (rs1056836) [25], *UGT1A6* Thr181Ala (rs1105879) [26], and *NAT2* Ile114Thr, Arg197Gln, Gly286Glu and Arg64Gln (rs1799930, rs1799931, rs1801279, rs180120) [27], in addition to two genes that had copy number variants, *GSTM1* and *GSTT1 [*[21]]. *NAT2* haplotypes were constructed using haplo.stats package in R (http://www.R-project.org/). *NAT2* haplotypes have been characterized for their impact on protein function [28, 29]; consistent with the existing classification [30], we classified carriers of two copies of the fast haplotype as “fast” and carriers of all other haplotypes as “slow” phenotype. All genotypes were obtained using Taqman assays, available “on demand” from ABI (Applied Biosystems, Foster City, CA), following manufacturer's instructions. Call rates were >97%. No differences were found between observed genotypic frequencies and those expected assuming Hardy–Weinberg Equilibrium.

### Statistical analyses

The analyses of questionnaire data were based on 761 localized cases, 1199 advanced cases, and 1139 controls. Analyses of genotype data were based on men with DNA from blood, including 535 localized cases, 988 advanced cases, and 800 controls. These individuals did not differ from those without DNA with regard to age, calorie intake, family history, SES and BMI at either study site (data not shown).

To best correct for differences in race/ethnicity, SES and the case/control ratio across the two study sites, we created a variable that classified men according to study site (SFBA or LAC), SES (5-level variable, as previously described [19]) and race/ethnicity (non-Hispanic White, AA, Hispanic), and used it to group individuals in conditional logistic regression models that were used to estimate odds ratios (OR) and 95% confidence intervals (CI). SES was collapsed into three categories (quintiles 1–2, 3, 4–5) for SFBA subjects and four categories (quintiles 1, 2, 3, 4–5) for LAC subjects, leaving six SES/race groups from SFBA and 12 from LAC. When evaluating smoking tobacco, models were adjusted for age at diagnosis for cases or selection into the study for controls (in years, modeled as continuous), family history of PCa in first-degree relatives (no, yes), BMI (<25.0, 25.0–29.9, ≥30.0 kg/m^2^), average lifetime consumption of alcohol (grams/day), use of nonsmoking tobacco (snuffing or chewing) (no, yes), cigar or pipe smoking (no, yes) if evaluating only cigarette smoking (cigarettes/day or pack-years), intake of red meat cooked at high temperature (broiled, pan-fried or grilled, in g/day), which we previously reported to be associated with increased PCa risk, and contributes to carcinogenic exposure [20, 31]. We also considered possible confounding by total vegetable consumption (g/day), total fruit consumption (g/day), and total calorie intake (kcal/day) during the reference year; however, inclusion of these covariates did not change OR estimates by >10%, so they were not included in final models. All analyses were stratified by stage (localized and advanced) and by race/ethnicity. Heterogeneity by race within each stage was evaluated using a likelihood ratio test comparing conditional logistic models that were fit with and without interaction terms of smoking variables and race.

#### Gene × smoking interaction analyses

We examined the potential modifying role of the selected polymorphisms on the associations between tobacco smoking and PCa risk using both two degree of freedom (2-df) interaction tests by treating the three-level tobacco smoking variables as categorical, and 1-df interaction tests by treating these variables as ordinal. We have previously reported the associations between these metabolic enzyme polymorphisms and PCa risk [20]. For the gene x environment analyses in this study, we evaluated one SNP for seven metabolism genes and two copy number variants (*GSTP1*, *PTGS2, CYP1A2, CYP2E1, EPHX1, CYP1B1, UGT1A6, GSTM1,* and *GSTT1*), as well as the predicted phenotype of the NAT2 enzyme determined by four SNPs in the gene used to define high and low enzymatic activity, as possible modifiers of the associations with the following smoking variables: smoking status (never, former, current), history of smoking tobacco (never, ever), age start of smoking tobacco (never smoker, ≤18 years, >18 years), smoking duration (never smoker, ≤29 years, >29 years), cigarettes smoked per day (never cigarette smoker, ≤20 cigarettes, >20 cigarettes), cigarette pack-years (never cigarette smoker, ≤22 cigarette pack-years, >22 cigarette pack-years), and years since quitting smoking (never smoker, >21 years, ≤21 years). Gene × smoking interaction models were adjusted for the same covariates used in the models to evaluate main effects of smoking on PCa risk.

All hypothesis tests were two sided and all analyses were done using the statistical software Stata S/E 11.2 (STATA Corporation, College Station, TX).

## Results

Socio-demographic and lifestyle characteristics, including tobacco smoking, of cases and controls are presented in Table 1. When compared to controls, localized and advanced cases were more likely to report a family history of PCa. Localized cases were of lower SES than controls. Among controls, 67% had ever smoked tobacco and 18% were current smokers during the reference year. They consumed, on average, about a pack of cigarettes a day and smoked for an average of 28.2 years. Tobacco smoking characteristics by PCa stage and race/ethnicity are presented in Table S1. No substantial differences in smoking characteristics were seen among races/ethnicities. Among controls, 65% of non-Hispanic Whites were ever tobacco smokers and smoked 30.7 pack-years compared to 73% of AA who smoked 27.4 pack-years and 70% of Hispanics who smoked 24.6 pack-years.

**Table 1:** Socio-demographic and lifestyle characteristics of controls and cases

	Controls (*N* = 1139)	Localized PCa cases (*N* = 761)	Advanced PCa cases (*N* = 1199)
	*n* (%)	*n* (%)	*n* (%)
Characteristics			
Age at diagnosis (years)			
<50	59 (5)	21 (3)	48 (4)
50–59	322 (29)	122 (16)	333 (28)
60–69	450 (40)	283 (38)	499 (42)
70+	293 (26)	323 (43)	310 (26)
*N*	1135	754	1195
Mean (SD)	63.7 (9.1)	67.5 (8.8)	63.9 (8.5)
Family history of PCa			
No	993 (88)	597 (79)	964 (81)
Yes	139 (12)	155 (21)	228 (19)
Body Mass Index (kg/m^2^)			
<25	290 (26)	199 (27)	294 (25)
25–29	514 (46)	374 (50)	579 (49)
≥30	320 (28)	176 (23)	317 (26)
Socio-economic status			
1 (Low)	124 (11)	161 (21)	161 (13)
2	142 (13)	136 (18)	150 (13)
3	206 (18)	127 (17)	217 (18)
4	278 (24)	138 (18)	235 (20)
5 (High)	385 (34)	192 (26)	432 (36)
Race/ethnicity			
Non-Hispanic White	764 (67)	343 (45)	741 (62)
African-American	249 (22)	277 (37)	255 (21)
Hispanic	122 (11)	134 (18)	199 (17)
Center			
SFBA	594 (52)	553 (73)	631 (53)
LAC	545 (48)	208 (27)	568 (47)
Ever smoked any tobacco			
Yes	763 (67)	560 (74)	839 (70)
Smoked cigarettes for at least 6 months			
Yes	707 (62)	531 (70)	782 (65)
Smoked cigars for at least 6 months			
Yes	148 (13)	109 (14)	159 (13)
Smoked pipes for at least 6 months			
Yes	198 (17)	127 (17)	220 (18)
Ever chewed tobacco			
Yes	21 (2)	18 (2)	28 (2)
Ever snuffed tobacco			
Yes	6 (1)	5 (1)	11 (1)
Tobacco smoking status (cigarettes/cigars/pipes)			
Never	369 (33)	197 (26)	357 (30)
Former	550 (49)	409 (55)	608 (51)
Current	209 (18)	143 (19)	228 (19)
Age start of smoking tobacco (years)			
*N*	759	552	835
Mean (SD)	18.5 (5.7)	18.4 (5.5)	18.3 (5.8)
Duration of smoking tobacco (years)			
*N*	759	552	835
Mean (SD)	28.2 (14.8)	32.0 (15.8)	28.9 (15.6)
Years passed since smoking cessation (former smokers only)			
*N*	549	407	603
Mean (SD)	21.2 (12.3)	22.1 (13.9)	22.8 (12.9)
Cigarettes smoked (per day)			
*N*	706	531	782
Mean (SD)	20.9 (14.5)	20.2 (14.9)	20.4 (14.8)
Cigarettes smoked (pack-years)			
*N*	701	525	778
Mean (SD)	29.1 (26.4)	32.3 (31.0)	29.2 (28.1)
Alcohol intake (g/day)			
*N*	1121	749	1188
Mean (SD)	12.0 (20.1)	12.9 (24.6)	12.4 (24.1)
Consumption of meat cooked at high temperature (g/day)			
*N*	1131	758	1194
Mean (SD)	119 (86)	140 (111)	129 (95)
Vegetable intake (g/day)			
*N*	1123	749	1189
Mean (SD)	137 (179)	145 (187)	134 (169)
Fruit intake (g/day)			
*N*	1123	749	1189
Mean (SD)	114 (184)	116 (173)	104 (165)
Daily caloric intake			
*N*	1096	717	1140
Mean (SD)	2627 (1079)	2845 (1137)	2853 (1137)

SFBA, San Francisco Bay Area; LAC, Los Angeles County.

Characteristics of PCa cases by smoking status (never smoker, quit >21 years ago, quit ≤21 years ago, current smoker) are presented in Table S2. When compared to never and former smokers, current smokers had a lower BMI (*P* = 0.002), lower SES (*P* = 0.001), were more likely to be non-Hispanic White or AA (*P* < 0.001), were of younger age at PCa diagnosis (*P* < 0.001), had higher levels of alcohol consumption during their lifetime (*P* < 0.001), consumed more meat cooked at high temperature (*P* < 0.001), had lower total vegetable consumption (*P* < 0.001), and lower total fruit intake (*P* < 0.001). When compared to former smokers, current smokers were more likely to smoke a pack or less (*P* < 0.001) and more likely to smoke for >29 years (*P* < 0.001).

### Tobacco smoking and prostate cancer risk

We observed differences in the associations between tobacco smoking variables and risk of localized PCa by race/ethnicity (Table 2). Among AA, there was no evidence of associations between localized PCa and any of the smoking variables. Among Hispanic and non-Hispanic Whites, ORs were generally elevated but were statistically significant only among non-Hispanic Whites. Among non-Hispanic Whites, risk of localized PCa was increased by 50% for ever smokers (OR = 1.5, 95% CI = 1.1–2.1), former smokers (OR = 1.5, 95% CI = 1.1–2.1), and current smokers (OR = 1.5; 95% CI = 0.9–2.4) compared to never smokers, although the last comparison was not statistically significant, probably due to the relatively small number of current smokers. OR estimates did not increase with increasing duration or intensity of smoking. Among former smokers, estimates were similar for men who quit >21 years (OR = 1.5; 95% CI = 1.0–2.1) vs. ≤21 years (OR = 1.6; 95% CI = 1.1–2.3) prior to the reference year.

**Table 2:** Smoking characteristics and risk of localized prostate cancer by race/ethnicity

	All races/ethnicities			Non-Hispanic Whites			African-Americans			Hispanics			
	Co/Ca	OR^1^	95% CI	Co/Ca	OR^1^	95% CI	Co/Ca	OR^1^	95% CI	Co/Ca	OR^1^	95% CI	Heterog p^2^
Smoking Status (any smoking tobacco)													
Never smoker	365/196	1.0^REF^		265/93	1.0^REF^		65/72	1.0^REF^		35/31	1.0^REF^		
Former smoker	549/409	1.3	1.0–1.6	385/200	1.5	1.1–2.1	111/132	0.8	0.5–1.3	53/77	1.6	0.9–3.2	0.073
Current smoker	206/142	1.1	0.8–1.5	108/47	1.5	0.9–2.4	68/72	0.7	0.4–1.2	30/23	1.1	0.5–2.3	
Use of smoking tobacco													
No	365/196	1.0^REF^		265/93	1.0^REF^		65/72	1.0^REF^		35/31	1.0^REF^		
Yes	756/553	1.3	1.0–1.6	494/248	1.5	1.1–2.1	179/204	0.8	0.5–1.2	83/101	1.4	0.8–2.8	0.057
Age at first tobacco use (years)													
Never smoker	365/196	1.0^REF^		265/93	1.0^REF^		65/72	1.0^REF^		35/31	1.0^REF^		
>18	292/217	1.3	1.0–1.7	187/100	1.5	1.0–2.0	78/82	0.7	0.4–1.2	27/35	1.4	0.6–3.2	
≤18	463/334	1.2	1.0–1.6	306/147	1.6	1.1–2.4	101/122	0.9	0.5–1.4	56/65	1.4	0.7–2.9	0.132
*p-trend*			0.106			0.040			0.640			0.354	
Smoking duration (years)													
Never smoker	365/196	1.0^REF^		265/93	1.0^REF^		65/72	1.0^REF^		35/31	1.0^REF^		
≤29	386/243	1.2	1.0–1.6	272/129	1.5	1.1–2.2	78/77	0.7	0.5–1.3	36/37	1.4	0.6–2.9	0.293
>29	369/308	1.3	1.0–1.6	221/118	1.5	1.1–2.8	101/127	0.8	0.5–1.3	47/63	1.5	0.7–3.0	
*p-trend*			0.081			0.022			0.476			0.293	
Cigarettes smoked per day													
	421/224	1.0^REF^		312/113	1.0^REF^		74/80	1.0^REF^		35/31	1.0^REF^		0.088
≤20	503/404	1.2	0.9–1.6	292/152	1.5	1.1–2.0	140/169	0.9	0.6–1.3	71/83	1.3	0.7–2.5	
>20	196/121	1.2	0.9–1.6	154/76	1.5	1.0–2.3	30/27	0.6	0.3–1.1	12/18	2.1	0.7–5.9	
*p-trend*			0.187			0.017			0.123			0.174	
Cigarette Pack-years													
Never cig. smoker	421/224	1.0^REF^		312/113	1.0^REF^		74/80	1.0^REF^		35/31	1.0^REF^		0.161
≤22	357/250	1.2	0.9–1.5	210/105	1.5	1.1–2.2	98/91	0.7	0.5–1.2	49/54	1.4	0.7–2.8	
>22	342/275	1.3	1.0–1.7	236/123	1.5	1.1–2.1	72/105	1.0	0.6–1.6	34/47	1.6	0.7–3.3	
*p-trend*			0.039			0.019			0.998			0.253	
Years since quitting smoking tobacco													
Never smoker	365/196	1.0^REF^		265/93	1.0^REF^		65	1.0^REF^		35/31	1.0^REF^		0.314
Quit >21 years ago	274/192	1.3	1.0–1.7	202/108	1.5	1.0–2.1	52	0.8	0.5–1.4	20/30	1.3	0.5–3.2	
Quit ≤21 years ago	274/215	1.3	1.0–1.7	183/91	1.6	1.1–2.3	58	0.9	0.5–1.5	33/46	1.8	0.8–3.8	
Current smoker	206/142	1.1	0.8–1.5	108/47	1.5	1.0–2.4	68	0.7	0.4–1.2	30/23	1.1	0.5–2.5	
*p-trend*			0.242			0.023			0.249			0.568	

1 Adjusted for age at diagnosis (years), family history of PCa, body mass index, alcohol consumption (g/day), total intake of meat cooked at high temperature (g/day), any lifetime use of nonsmoking tobacco snuff/chew, use of cigar/pipe for at least 6 months if evaluating cigarette smoking (per day and pack-years).

2 Test of heterogeneity of ORs by race/ethnicity.

Table 3 presents associations between smoking variables and risk of advanced PCa stratified by race/ethnicity. Among non-Hispanic Whites, current smoking was associated with an increased risk of advanced PCa when compared to never smokers (OR = 1.4, 95% CI = 1.0–1.9). No associations were observed among AA (OR = 0.8, 95% CI = 0.5–1.3) or Hispanics (OR = 0.5, 95% CI = 0.2–1.0; p of heterogeneity test = 0.004). OR estimates did not increase with increasing duration or intensity of smoking. Among Hispanics, compared to never smokers, we observed some borderline significant associations for smokers with longer time since quitting and an inverse association with current smoking, although the number of current smokers was relatively small.

**Table 3:** Smoking characteristics and risk of advanced prostate cancer by race/ethnicity

	All races/ethnicities			Non-Hispanic Whites			African-Americans			Hispanics			
	Co/Ca	OR^1^	95% CI	Co/Ca	OR^1^	95% CI	Co/Ca	OR^1^	95% CI	Co/Ca	OR^1^	95% CI	Heterog p^2^
Smoking Status (any smoking tobacco)													
Never smoker	365/355	1.0^REF^		265/228	1.0^REF^		65/67	1.0^REF^		35/60	1.0^REF^		
Former smoker	549/606	1.1	0.9–1.3	385/381	1.1	0.9–1.4	111/110	0.8	0.5–1.2	53/115	1.3	0.8–2.3	0.004
Current smoker	206/227	1.1	0.9–1.4	108/130	1.4	1.0–1.9	68/75	0.8	0.5–1.3	30/22	0.5	0.2–1.0	
Use of smoking tobacco													
No	365/355	1.0^REF^		265/228	1.0^REF^		65/67	1.0^REF^		35/60	1.0^REF^		
Yes	756/833	1.1	0.9–1.3	494/511	1.2	0.9–1.5	179/185	0.8	0.5–1.2	83/137	1.0	0.6–1.7	0.359
Age at first tobacco use (years)													
Never smoker	365/355	1.0^REF^		265/228	1.0^REF^		65/67	1.0^REF^		35/60	1.0^REF^		
>18	292/309	1.1	0.8–1.3	187/199	1.2	0.9–1.6	78/69	0.7	0.4–1.1	27/41	0.9	0.5–1.8	
≤18	463/523	1.1	0.9–1.4	306/312	1.2	0.9–1.5	101/116	0.9	0.6–1.4	56/95	1.0	0.6–1.8	0.474
*p-trend*			0.262			0.178			0.884			0.929	
Smoking duration (years)													
Never smoker	365/355	1.0^REF^		265/228	1.0^REF^		65/67	1.0^REF^		35/60	1.0^REF^		
≤29	386/404	1.1	0.9–1.3	272/255	1.1	0.9–1.4	78/72	0.8	0.5–1.3	36/77	1.3	0.7–2.5	0.063
>29	369/428	1.1	0.9–1.4	221/256	1.3	1.0–1.7	101/113	0.8	0.5–1.3	47/59	0.7	0.4–1.3	
*p-trend*			0.275			0.054			0.464			0.225	
Cigarettes smoked per day													
Never cig. smoker	421/412	1.0^REF^		312/278	1.0^REF^		74/72	1.0^REF^		35/62	1.0^REF^		
≤20	503/576	1.1	0.9–1.3	292/304	1.1	0.9–1.4	140/153	0.9	0.6–1.4	71/119	1.0	0.6–1.7	0.801
>20	196/200	1	0.8–1.3	154/157	1.1	0.8–1.5	30/27	0.7	0.4–1.4	16–Dec	0.8	0.3–2.0	
*p-trend*			0.763			0.496			0.344			0.652	
Cigarette Pack-years													
Never cig. smoker	421/412	1.0^REF^		312/278	1.0^REF^		74/72	1.0^REF^		35/62	1.0^REF^		
≤22	357/386	1	0.8–1.2	210/194	1.0	0.8–1.3	98/100	0.9	0.5–1.4	49/92	1.1	0.6–1.9	0.256
>22	342/390	1.1	0.9–1.4	236/267	1.2	0.9–1.6	72/80	0.9	0.5–1.4	34/43	0.7	0.4–1.3	
*p-trend*			0.351			0.142			0.594			0.303	
Years since quitting smoking tobacco													
Never smoker	365	1.0^REF^		265/228	1.0^REF^		65/74	1.0^REF^		35/66	1.0^REF^		
Quit >21 years ago	274	1.1	0.9–1.4	202/207	1.2	0.9–1.5	52/42	0.6	0.4–1.1	20/69	2.0	1.0–4.0	<0.001
Quit ≤21 years ago	274	1	0.8–1.3	183/171	1.0	0.8–1.4	58/74	0.9	0.5–1.5	33/60	1.0	0.5–1.8	
Current smoker	206	1.1	0.9–1.4	108/130	1.4	1.0–1.9	68/85	0.8	0.5–1.3	30/24	0.5	0.2–1.0	
*p-trend*			0.508			0.114			0.616			0.044	

1 Adjusted for age at diagnosis (years), family history of PCa, body mass index, alcohol consumption (g/day), total intake of meat cooked at high temperature (g/day), any lifetime use of nonsmoking tobacco snuff/chew, use of cigar/pipe for at least 6 months if evaluating cigarette smoking (per day and pack-years).

2 Test of heterogeneity of ORs by race/ethnicity.

When restricting our analyses to ever smokers, we examined whether the age at first tobacco use modified the associations between tobacco smoking duration, cigarette pack-years, and smoking status (quit >21 years ago, quit ≤21 years ago, current smoking) and PCa risk. There was no evidence of effect modification for either localized or advanced disease among the variables considered (data not shown).

### Tobacco smoking, polymorphisms in metabolism enzymes, and PCa risk

Interactions between each of the nine polymorphisms and NAT2 predicted phenotype and tobacco smoking variables were evaluated. We only observed evidence of effect modification for *CYP1A2* -154A>C (rs762551) on smoking status (never, former, current) (Table 4). Among carriers of the CC genotype, current smoking was associated with increased risk of PCa overall (OR = 2.2; 95% CI = 1.2–4.3, *P*-interaction = 0.008), localized PCa (OR = 2.8; 95% CI = 1.2–6.9, *P*-interaction = 0.012), and advanced PCa (OR = 1.9; 95% CI = 1.0–3.8, *P*-interaction = 0.043). These associations were not present among carriers of the AA genotype. Analyses considering other smoking variables (smoking duration, cigarette pack-years, and age at first tobacco use) showed similar findings as those for smoking status; however, none reached statistical significance. Similar interaction ORs were observed when stratifying by race/ethnicity and including both localized and advanced PCa for non-Hispanic Whites and AA (data not shown). This pattern was not observed among Hispanics, although the number of Hispanics was small (data not shown). No evidence of interaction was observed for any of the other polymorphisms or NAT2 predicted phenotype. We also conducted exploratory analyses to consider all polymorphisms and NAT2 predicted phenotype jointly using principal components analyses. We found no evidence that components defined by multiple polymorphism modified the association between smoking and PCa risk (data not shown).

**Table 4:** Smoking status, CYP1A2 (*rs7662551*) genotype, and risk of prostate cancer by cancer stage

CYP1A2	A/A				A/C				C/C				*P*-interaction
All cases													
Smoking status	Co	Ca	OR^1^	95% CI	Co	Ca	OR^1^	95% CI	Co	Ca	OR^1^	95% CI	
Never	126	224	1.0^REF^		102	159	1.0^REF^		23	40	1.0^REF^		0.008
Former	170	382	1.2	0.9–1.6	164	319	1.0	0.8–1.3	42	63	1.0	0.6–1.5	
Current	88	136	0.9	0.6–1.2	52	121	1.4	1.0–2.0	10	36	2.2	1.2–4.3	
*p-trend*				0.601				0.066				0.031	
Localized cases													
Smoking status	Co	Ca	OR^1^	95% CI	Co	Ca	OR^1^	95% CI	Co	Ca	OR^1^	95% CI	
Never	126	70	1.0^REF^		102	55	1.0^REF^		23	13	1.0^REF^		0.016
Former	170	131	1.4	1.0–2.2	164	120	1.2	0.9–1.7	42	25	1.0	0.5–2.2	
Current	88	39	0.8	0.5–1.4	52	49	1.5	0.9–2.4	10	16	2.8	1.1–6.9	
*p-trend*				0.710				0.075				0.038	
Advanced cases													
Smoking status	Co	Ca	OR^1^	95% CI	Co	Ca	OR^1^	95% CI	Co	Ca	OR^1^	95% CI	
Never	126	154	1.0^REF^		102	104	1.0^REF^		23	27	1.0^REF^		0.043
Former	170	251	1.1	0.8–1.5	164	199	1.0	0.7–1.3	42	38	0.9	0.5–1.5	
Current	88	97	0.9	0.8–1.5	52	72	1.3	0.9–1.9	10	20	1.9	1.0–3.8	
*p-trend*				0.620				0.238				0.148	

1 Odds ratios derived from a common baseline model that includes the genotype, smoking status, and interaction terms between genotype and smoking status. ORs are adjusted for age at diagnosis (years), family history of PCa, body mass index, alcohol consumption (g/day), total intake of meat cooked at high temperature (g/day), any lifetime use of nonsmoking tobacco snuff/chew.

## Discussion

In this study, ever smoking was found to be associated with localized PCa risk, particularly among non-Hispanic Whites. Quitting smoking was also associated with localized PCa. In contrast, being a current smoker was associated with risk of advanced PCa among non-Hispanic White men. For both localized and advanced PCa, the association with smoking was modified by a polymorphism in the carcinogen metabolism *CYP1A2* gene. Overall, our findings lend support to a role for tobacco smoking in PCa risk after taking into account both PCa stage and race/ethnicity in the analyses.

In congruence with our findings, a population-based case–control study in the U.S. reported that current cigarette smoking was associated with an increased risk of PCa when compared to non-smoking [32]. In that study, PCa risk increased with increasing pack-years of cigarette smoking, something we did not observe in our study. Moreover, in contrast with our study, a stronger association was observed between pack-years and aggressive PCa, and quitting smoking was associated with reduced PCa risk. However, our observations of former smokers having an increased risk of localized PCa, and non-Hispanic White current smokers having an increased risk of advanced PCa, are consistent with findings from a 2010 meta-analysis of 24 prospective cohort studies showing that both former and current smokers had an increased risk of incident PCa, although stage and race/ethnicity were not accounted for in the meta-analysis [1]. A large cohort study including data from 10 European countries (EPIC), which considered stage and grade, reported an inverse association between localized and low-grade prostate cancer (PCa) among smokers, which is in contrast with our results that showed a positive association [33]. This study also reported no significant association with smoking among advanced and high-grade cases. In contrast, the Japan Public Health Center-based prospective study (JPHC study), which included over 48,000 men, and a study using the Shared Equal Access Regional Cancer Hospital (SEARCH) cohort both found a positive association between smoking and diagnosis of advanced PCa [34, 35]. Based on the available literature the 2014 Surgeon General's Report on smoking and tobacco use concluded that there is suggestive evidence showing smoking to be a risk factor of being diagnosed with advanced stage or high-grade PCa, which is a risk factor for progression and death [36]. Similar to 2010 meta-analyses mentioned above, this report highlighted the scarcity of studies that took into account stage and grade at diagnosis.

As with other tobacco-related cancers, a possible mechanism by which tobacco smoking might influence PCa risk is the action of tobacco-related carcinogens that could induce DNA damage in the prostate. These mutagenic carcinogens can be endogenously metabolized to their active forms, which upon reaching the target tissues can bind to DNA. Alternatively, they can be detoxified to less active forms that can be readily excreted from the body. Carcinogen metabolism enzymes are responsible for the detoxification or activation of mutagenic carcinogens and are coded by genes known to be variable in the population [37]. In this study, we found that the association between smoking status and PCa risk was modified by *CYP1A2* -154A>C (rs762551), a gene that codes for an enzyme that plays a key role in the metabolism of many drugs, such as caffeine, and in the activation of various tobacco carcinogens, including HCAs and PAHs [38–42]. We observed that the association between current smoking status and PCa risk seemed restricted to carriers of one or two copies of the C allele. CYP1A2 is an inducible phase I metabolizing enzyme highly active in the liver, where it plays a predominant role in the activation of HCAs [40], such as those found in tobacco, to reactive species that can undergo further activation in the liver or detoxification. *CYP1A2* mRNA is also expressed in prostate tissue [43–45]. *CYP1A2* expression is variable in the general population and the *CYP1A2* -154A>C polymorphism may partially explain the observed variability in *CYP1A2* inducibility, with the protein coded by the A allele being correlated with higher enzymatic activity than the one coded by the C allele [42]. We have previously reported that the association between well-done cooked red meat, known to accumulate HCAs, and colorectal cancer was stronger among carriers of the C allele [46]. A meta-analysis of 19 case–control studies further showed that the CC genotype is associated with an increased risk of various types of cancer combined (including breast, colorectal, lung, pancreatic, ovarian, stomach, and bladder) and a significant increase in risk among Caucasians [47]. Furthermore, other studies showed that low activity of *CYP1A2* was associated with risk of testicular cancer [48] and PCa [49]. A possible explanation for these findings is that slower activation of HCAs in the liver by *CYP1A2* may allow HCAs to remain in the body's circulation longer, which could result in greater amounts of HCAs reaching other organs and tissues, such as the prostate. We cannot exclude, however, the possibility that our finding might be a false positive; Bonferroni adjustment of the *CYP1A2* -154A>C by smoking interaction *P*-value by the number of SNPs/phenotypes tested (*n* = 10) would render an interaction *P*-value of borderline significance (*P* = 0.08). Since *CYP1A2* is heavily involved in caffeine metabolism, we also considered frequency of coffee intake in the interaction models to take into account possible confounding, but ORs did not change by >10%.

Screening bias has been identified as a possible limitation in previous studies, and could be present if tobacco smoking patterns were correlated with PCa screening, specifically prostate-specific antigen (PSA) screening [3]. We evaluated potential confounding by PSA screening during the 5 years prior to the reference year among the cases and controls from the SFBA study site, for whom we had data on PSA screening. Including PSA screening in the model with other covariates did not change the OR estimates for any of the smoking variables by >10% and was therefore not included in the final model. Furthermore, there was no statistical difference in PSA screening when comparing cases with controls: 76% of controls, 80% of localized cases, and 71% of advanced cases reported previous PSA screening.

The overall strengths of this study include the utilization of a population-based study design with cases obtained from two SEER cancer registries, a large sample size of cases and controls, a multiethnic population that includes non-Hispanic White, AA, and Hispanic men, oversampling of advanced cases, and detailed information on lifestyle and other characteristics. Another strength is the consideration of genetic variation in tobacco carcinogen metabolism enzymes to examine gene by environment interactions. Among the limitations of this study is the inclusion of only a few selected functional SNPs for each candidate gene, which did not allow us to comprehensively consider all their genetic variation. Other limitations include the lack of data on environmental tobacco exposure, which may have introduced some exposure misclassification and finally, small sample sizes when stratifying the analyses by multiple factors (stage, race/ethnicity, age at diagnosis), which reduced our statistical power to detect possible associations.

In summary, our findings support a role for tobacco smoking and risk of PCa, and suggest that ever smokers, including those who quit, are at increased risk of having localized PCa, whereas current smokers have a statistically significant increased risk of advanced PCa. Moreover, our gene-environment analyses support a role for tobacco carcinogens in PCa risk, further strengthening an association between tobacco smoke and PCa risk.

## Supporting Information

**Table S1.** Tobacco smoking characteristics by race/ethnicity among cases and controls.

**Table S2.** Characteristics of prostate cancer cases by tobacco smoking status (including any cigarette/cigar/pipe smoking).
