# Supplementary material for: Tobacco smoking, polymorphisms in carcinogen metabolism enzyme genes, and risk of localized and advanced prostate cancer: results from the California Collaborative Prostate Cancer Study
Source: Cancer Med. 2014 Oct 30;3(6):1644–55. doi: 10.1002/cam4.334 (PMC4298391; doi:10.1002/cam4.334)
Supplement: Supplementary file 1 — Table S1. Tobacco smoking characteristics by race/ethnicity among cases and controls. [file cam40003-1644-sd1.docx]

| **Supplementary table 1. Tobacco smoking characteristics by race/ethnicity among cases and controls** | | | | | | | | | | | | | | | | | | | | | | | | | | | | | | | | | | | | | | | | | | | | | | | |  | | | |  |  |
| --- | --- | --- | --- | --- | --- | --- | --- | --- | --- | --- | --- | --- | --- | --- | --- | --- | --- | --- | --- | --- | --- | --- | --- | --- | --- | --- | --- | --- | --- | --- | --- | --- | --- | --- | --- | --- | --- | --- | --- | --- | --- | --- | --- | --- | --- | --- | --- | --- | --- | --- | --- | --- | --- |
|  | **Controls** | | | | | | | | | | | | |  | | **Localized PCa cases** | | | | | | | | | | | | | | | |  | | **Advanced PCa cases** | | | | | | | | | | | | | |  | | | |  |  |
|  | Non-Hispanic Whites | | | African-Americans | | | | | | Hispanics | | | |  | | Non-Hispanic Whites | | | | | | African-Americans | | | | | | Hispanics | | | |  | | Non-Hispanic Whites | | | | African-Americans | | | | | Hispanics | | | | | | | |  |  |  |
|  | n | % |  | | n | | % | |  | | n | | % | |  | | n | | % | |  | | n | | % | |  | | n | | % | |  | | n | | % | |  | | n | % | | |  | | n | | | % | | | |
| **Smoked cigarettes for at least 6 months** | | |  | |  | |  | |  | |  | |  | |  | |  | |  | |  | |  | |  | |  | |  | |  | |  | |  | |  | |  | |  |  | | |  | |  | | |  | | | |
| **No** | 313 | 41 |  | | 75 | | 30 | |  | | 36 | | 30 | |  | | 113 | | 33 | |  | | 80 | | 29 | |  | | 31 | | 23 | |  | | 278 | | 38 | |  | | 73 | 29 | | |  | | 62 | | | 31 | | | |
| **Yes** | 449 | 59 |  | | 172 | | 70 | |  | | 83 | | 70 | |  | | 228 | | 67 | |  | | 197 | | 71 | |  | | 101 | | 77 | |  | | 461 | | 62 | |  | | 182 | 71 | | |  | | 136 | | | 69 | | | |
|  |  |  |  | |  | |  | |  | |  | |  | |  | |  | |  | |  | |  | |  | |  | |  | |  | |  | |  | |  | |  | |  |  | | |  | |  | | |  | | | |
| **Smoked cigars for at least 6 months** | |  |  | |  | |  | |  | |  | |  | |  | |  | |  | |  | |  | |  | |  | |  | |  | |  | |  | |  | |  | |  |  | | |  | |  | | |  | | | |
| **No** | 648 | 85 |  | | 219 | | 89 | |  | | 114 | | 96 | |  | | 286 | | 84 | |  | | 232 | | 84 | |  | | 124 | | 94 | |  | | 621 | | 84 | |  | | 224 | 88 | | |  | | 190 | | | 96 | | | |
| **Yes** | 114 | 15 |  | | 28 | | 11 | |  | | 5 | | 5 | |  | | 55 | | 16 | |  | | 45 | | 16 | |  | | 8 | | 6 | |  | | 118 | | 16 | |  | | 31 | 12 | | |  | | 8 | | | 4 | | | |
|  |  |  |  | |  | |  | |  | |  | |  | |  | |  | |  | |  | |  | |  | |  | |  | |  | |  | |  | |  | |  | |  |  | | |  | |  | | |  | | | |
| **Smoked pipes for at least 6 months** | |  |  | |  | |  | |  | |  | |  | |  | |  | |  | |  | |  | |  | |  | |  | |  | |  | |  | |  | |  | |  |  | | |  | |  | | |  | | | |
| **No** | 599 | 79 |  | | 215 | | 87 | |  | | 116 | | 97 | |  | | 260 | | 76 | |  | | 234 | | 85 | |  | | 129 | | 98 | |  | | 562 | | 76 | |  | | 217 | 85 | | |  | | 194 | | | 98 | | | |
| **Yes** | 163 | 21 |  | | 32 | | 13 | |  | | 3 | | 3 | |  | | 81 | | 24 | |  | | 43 | | 15 | |  | | 3 | | 2 | |  | | 177 | | 24 | |  | | 38 | 15 | | |  | | 4 | | | 2 | | | |
|  |  |  |  | |  | |  | |  | |  | |  | |  | |  | |  | |  | |  | |  | |  | |  | |  | |  | |  | |  | |  | |  |  | | |  | |  | | |  | | | |
| **Ever chewed tobacco** |  |  |  | |  | |  | |  | |  | |  | |  | |  | |  | |  | |  | |  | |  | |  | |  | |  | |  | |  | |  | |  |  | | |  | |  | | |  | | | |
| **No** | 750 | 98 |  | | 241 | | 98 | |  | | 117 | | 98 | |  | | 336 | | 99 | |  | | 266 | | 96 | |  | | 130 | | 98 | |  | | 719 | | 97 | |  | | 247 | 97 | | |  | | 198 | | | 100 | | | |
| **Yes** | 12 | 2 |  | | 6 | | 2 | |  | | 2 | | 2 | |  | | 5 | | 1 | |  | | 11 | | 4 | |  | | 2 | | 2 | |  | | 20 | | 3 | |  | | 8 | 3 | | |  | | 0 | | | 0 | | | |
|  |  |  |  | |  | |  | |  | |  | |  | |  | |  | |  | |  | |  | |  | |  | |  | |  | |  | |  | |  | |  | |  |  | | |  | |  | | |  | | | |
| **Ever snuffed tobacco** |  |  |  | |  | |  | |  | |  | |  | |  | |  | |  | |  | |  | |  | |  | |  | |  | |  | |  | |  | |  | |  |  | | |  | |  | | |  | | | |
| **No** | 758 | 99 |  | | 245 | | 99 | |  | | 119 | | 100 | |  | | 338 | | 99 | |  | | 275 | | 99 | |  | | 132 | | 100 | |  | | 729 | | 99 | |  | | 254 | 99 | | |  | | 198 | | | 100 | | | |
| **Yes** | 4 | 1 |  | | 2 | | 1 | |  | | 0 | | 0 | |  | | 3 | | 1 | |  | | 2 | | 1 | |  | | 0 | | 0 | |  | | 10 | | 1 | |  | | 1 | 1 | | |  | | 0 | | | 0 | | | |
|  |  |  |  | |  | |  | |  | |  | |  | |  | |  | |  | |  | |  | |  | |  | |  | |  | |  | |  | |  | |  | |  |  | | |  | |  | | |  | | | |
| **Ever smoked any tobacco** |  |  |  | |  | |  | |  | |  | |  | |  | |  | |  | |  | |  | |  | |  | |  | |  | |  | |  | |  | |  | |  |  | | |  | |  | | |  | | | |
| **No** | 266 | 35 |  | | 66 | | 27 | |  | | 36 | | 30 | |  | | 93 | | 27 | |  | | 72 | | 26 | |  | | 31 | | 23 | |  | | 228 | | 31 | |  | | 68 | 27 | | |  | | 60 | | | 30 | | | |
| **Yes** | 496 | 65 |  | | 181 | | 73 | |  | | 83 | | 70 | |  | | 248 | | 73 | |  | | 205 | | 74 | |  | | 101 | | 77 | |  | | 551 | | 69 | |  | | 187 | 73 | | |  | | 138 | | | 70 | | | |
|  |  |  |  | |  | |  | |  | |  | |  | |  | |  | |  | |  | |  | |  | |  | |  | |  | |  | |  | |  | |  | |  |  | | |  | |  | | |  | | | |
| **Smoking Status** |  |  |  | |  | |  | |  | |  | |  | |  | |  | |  | |  | |  | |  | |  | |  | |  | |  | |  | |  | |  | |  |  | | |  | |  | | |  | | | |
| **Never** | 266 | 35 |  | | 66 | | 27 | |  | | 36 | | 30 | |  | | 93 | | 27 | |  | | 72 | | 26 | |  | | 31 | | 24 | |  | | 228 | | 31 | |  | | 68 | 27 | | |  | | 60 | | | 30 | | | |
| **Former** | 385 | 51 |  | | 112 | | 45 | |  | | 53 | | 45 | |  | | 200 | | 59 | |  | | 132 | | 48 | |  | | 77 | | 49 | |  | | 381 | | 52 | |  | | 111 | 43 | | |  | | 116 | | | 59 | | | |
| **Current** | 110 | 14 |  | | 69 | | 28 | |  | | 30 | | 25 | |  | | 47 | | 14 | |  | | 73 | | 26 | |  | | 23 | | 17 | |  | | 130 | | 57 | |  | | 76 | 30 | | |  | | 22 | | | 11 | | | |
|  |  |  |  | |  | |  | |  | |  | |  | |  | |  | |  | |  | |  | |  | |  | |  | |  | |  | |  | |  | |  | |  |  | | |  | |  | | |  | | | |
| **Age start of smoking tobacco (years of age)** | | |  | |  | |  | |  | |  | |  | |  | |  | |  | |  | |  | |  | |  | |  | |  | |  | |  | |  | |  | |  |  | | |  | |  | | |  | | | |
| **N** | 495 |  |  | | 181 | |  | |  | | 83 | |  | |  | | 247 | |  | |  | | 205 | |  | |  | | 100 | |  | |  | | 511 | |  | |  | | 187 |  | | |  | | 137 | | |  | | | |
| **Mean(SD)** | 18.6 (6.1) |  |  | | 18.5 (4.9) | |  | |  | | 17.6 (5.3) | | | |  | | 18.7 (5.3) | |  | |  | | 18.4 (6.1) | | | |  | | 17.8 (4.9) | | | |  | | 18.5 (5.6) | | | |  | | 17.9 (6.0) |  | | |  | | 18.0 (6.4) | | | | | | |
|  |  |  |  | |  | |  | |  | |  | |  | |  | |  | |  | |  | |  | |  | |  | |  | |  | |  | |  | |  | |  | |  |  | | |  | |  | | |  | | | |
| **Duration of smoking tobacco (years)** | |  |  | |  | |  | |  | |  | |  | |  | |  | |  | |  | |  | |  | |  | |  | |  | |  | |  | |  | |  | |  |  | | |  | |  | | |  | | | |
| **N** | 495 |  |  | | 181 | |  | |  | | 83 | |  | |  | | 247 | |  | |  | | 205 | |  | |  | | 100 | |  | |  | | 511 | |  | |  | | 187 |  | | |  | | 137 | | |  | | | |
| **Mean(SD)** | 27.0 (14.9) |  |  | | 30.8 (14.4) | | | |  | | 29.4 (14.3) | | | |  | | 29.5 (15.5) | | | |  | | 34.1 (15.6) | | | |  | | 34.1 (16.2) | | | |  | | 28.1 (15.4) | | | |  | | 32.8 (14.7) | | | |  | | 26.3 (16.7) | | | | | | |
|  |  |  |  | |  | |  | |  | |  | |  | |  | |  | |  | |  | |  | |  | |  | |  | |  | |  | |  | |  | |  | |  |  | | |  | |  | | |  | | | |
| **Years passed since smoking cessation (former smokers only)** | | | | | |  | |  | |  | |  | |  | |  | |  | |  | |  | |  | |  | |  | |  | |  | |  | |  | |  | |  | |  | |  | |  | | |  | | | |  |
| **N** | 385 |  |  | | 111 | |  | |  | | 53 | |  | |  | | 199 | |  | |  | | 132 | |  | |  | | 76 | |  | |  | | 378 | |  | |  | | 109 |  | | |  | | 116 | | |  | | | |
| **Mean(SD)** | 22.0 (12.0) |  |  | | 20.1 (12.3) | | | |  | | 18.2 (13.0) | | | |  | | 24.2(13.7) | | | |  | | 19.8 (13.2) | | | |  | | 20.4 (14.9) | | | |  | | 23.7 (12.9) | | | |  | | 18.9 (11.1) | | | |  | | 23.5 (13.8) | | | | | | |
|  |  |  |  | |  | |  | |  | |  | |  | |  | |  | |  | |  | |  | |  | |  | |  | |  | |  | |  | |  | |  | |  |  | | |  | |  | | |  | | | |
| **Cigarettes smoked (pack-years)** | |  |  | |  | |  | |  | |  | |  | |  | |  | |  | |  | |  | |  | |  | |  | |  | |  | |  | |  | |  | |  |  | | |  | |  | | |  | | | |
| **N** | 448 |  |  | | 172 | |  | |  | | 83 | |  | |  | | 228 | |  | |  | | 196 | |  | |  | | 101 | |  | |  | | 461 | |  | |  | | 181 |  | | |  | | 136 | | |  | | | |
| **Mean(SD)** | 30.7 (27.1) |  |  | | 27.4 (25.4) | | | |  | | 24.6 (24.4) | | | |  | | 35.2 (34.6) | | | |  | | 29.5 (26.0) | | | |  | | 31.1 (31.0) | | | |  | | 33.4 (29.6) | | | |  | | 26.1 (25.3) | | | |  | | 19.2 (23.3) | | | | | | |
|  |  |  |  | |  | |  | |  | |  | |  | |  | |  | |  | |  | |  | |  | |  | |  | |  | |  | |  | |  | |  | |  |  | | |  | |  | | |  | | | |
| **Cigarettes smoked (per day)** | |  |  | |  | |  | |  | |  | |  | |  | |  | |  | |  | |  | |  | |  | |  | |  | |  | |  | |  | |  | |  |  | | |  | |  | | |  | | | |
| **N** | 448 |  |  | | 172 | |  | |  | | 83 | |  | |  | | 228 | |  | |  | | 197 | |  | |  | | 101 | |  | |  | | 461 | |  | |  | | 182 |  | | |  | | 136 | | |  | | | |
| **Mean(SD)** | 23.2 (14.5) |  |  | | 17.7 (13.9) | | | |  | | 15.6 (12.9) | | | |  | | 24.2 (16.5) | | | |  | | 17.0 (12.1) | | | |  | | 17.7 (14.2) | | | |  | | 23.6 (14.7) | | | |  | | 17.1 (14.8) | | | |  | | 14.2 (12.3) | | | | | | |
|  |  |  |  | |  | |  | |  | |  | |  | |  | |  | |  | |  | |  | |  | |  | |  | |  | |  | |  | |  | |  | |  |  | | |  | |  | | |  | | | |
