# Supplementary material for: Tobacco smoking, polymorphisms in carcinogen metabolism enzyme genes, and risk of localized and advanced prostate cancer: results from the California Collaborative Prostate Cancer Study
Source: Cancer Med. 2014 Oct 30;3(6):1644–55. doi: 10.1002/cam4.334 (PMC4298391; doi:10.1002/cam4.334)
Supplement: Supplementary file 2 — Table S2. Characteristics of prostate cancer cases by tobacco smoking status (including any cigarette/cigar/pipe smoking). [file cam40003-1644-sd2.docx]

| **Supplementary Table 2.** Characteristics of prostate cancer cases by tobacco smoking status (including any cigarette/cigar/pipe smoking) | | | | | |
| --- | --- | --- | --- | --- | --- |
|  | **Never smoker** | **Former Smoker** | | **Current smoker** |  |
|  |  | **Quit >21 years ago** | **Quit ≤21 years ago** |  |  |
|  | n (%) | n (%) | n (%) | n (%) | p-value |
|  |  |  |  |  |  |
| **Age at diagnosis (years)** | |  |  |  |  |
| <50 | 27 (5) | 5 (1) | 14 (3) | 23 (6) | <0.001 |
| 50-59 | 137 (25) | 74 (14) | 121 (24) | 123 (34) |  |
| 60-69 | 217 (39) | 214 (42) | 201 (41) | 146 (39) |  |
| 70+ | 170 (31) | 222 (43) | 158 (32) | 78 (21) |  |
|  |  |  |  |  |  |
| N | 552 | 515 | 495 | 371 | <0.001 |
| Mean (SD) | 65 (9) | 68 (8) | 65 (8) | 62 (8) |  |
|  |  |  |  |  |  |
| **Stage** |  |  |  |  | 0.273 |
| Localized | 197 (36) | 202 (39) | 205 (41) | 143 (39) |  |
| Advanced | 357 (64) | 313 (61) | 290 (59) | 228 (62) |  |
|  |  |  |  |  |  |
| **Family history of PCa** |  |  |  |  |  |
| No | 447 (81) | 405 (79) | 390 (79) | 309 (83) | 0.278 |
| Yes | 105 (19) | 110 (21) | 105 (21) | 62 (17) |  |
|  |  |  |  |  |  |
| **Body Mass Index (kg/m^2^)** |  |  |  |  |  |
| <25 | 133 (24) | 141 (27) | 100 (20) | 118 (32) | 0.002 |
| 25-29 | 269 (49) | 256 (50) | 251 (51) | 175 (47) |  |
| ≥30 | 152 (27) | 118 (23) | 143 (29) | 77 (21) |  |
|  |  |  |  |  |  |
| **Socio-economic status** |  |  |  |  |  |
| 1(Low) | 86 (16) | 62 (12) | 96 (19) | 74 (20) | 0.001 |
| 2 | 62 (11) | 76 (15) | 82 (17) | 63 (17) |  |
| 3 | 89 (16) | 91 (18) | 89 (18) | 73 (20) |  |
| 4 | 124 (21) | 99 (19) | 85 (17) | 63 (17) |  |
| 5(High) | 191 (36) | 187 (36) | 143 (29) | 98 (26) |  |
|  |  |  |  |  |  |
| **Race /ethnicity** |  |  |  |  |  |
| Non-Hispanic White | 321 (58) | 318 (62) | 259 (52) | 177 (48) | <0.001 |
| African-American | 140 (25) | 97 (19) | 144 (29) | 149 (40) |  |
| Hispanic | 91 (17) | 100 (19) | 92 (19) | 45 (12) |  |
|  |  |  |  |  |  |
| **Center** |  |  |  |  |  |
| SFBA | 348 (62) | 303 (59) | 302 (61) | 218 (59) | 0.567 |
| LAC | 209 (38) | 212 (41) | 193 (39) | 153 (41) |  |
|  |  |  |  |  |  |
| **Age start smoking tobacco (years)** | | |  |  |  |
| >18 | n/a | 209 (41) | 188 (38) | 129 (35) | 0.204 |
| ≤18 | n/a | 305 (59) | 307 (62) | 242 (65) |  |
|  |  |  |  |  |  |
| **Duration of smoking tobacco (years)** | | |  |  |  |
| ≤29 | n/a | 464 (90) | 145 (29) | 35 (9) | <0.001 |
| >29 | n/a | 50 (10) | 350 (71) | 336 (91) |  |
|  |  |  |  |  |  |
| **Cigarettes smoked (per day)** | |  |  |  |  |
| ≤20 | n/a | 369 (77) | 312 (67) | 294 (84) | <0.001 |
| >20 | n/a | 108 (23) | 156 (33) | 58 (16) |  |
|  |  |  |  |  |  |
| **Cigarette Pack-years** |  |  |  |  |  |
| *≤22* | n/a | 343 (72) | 179 (38) | 110 (31) | <0.001 |
| *>22* | n/a | 134 (28) | 288 (62) | 241 (69) |  |
|  |  |  |  |  |  |
| **Alcohol intake (g/day)** |  |  |  |  |  |
| N | 551 | 514 | 494 | 369 | <0.001 |
| Mean (SD) | 7 (16) | 12 (22) | 14 (28) | 20 (30) |  |
|  |  |  |  |  |  |
| **Meat cooked at high temperature intake (g/day)** | | | |  |  |
| N | 552 | 514 | 494 | 371 | 0.002 |
| Mean (SD) | 57 (52) | 51 (51) | 60 (56) | 65 (55) |  |
|  |  |  |  |  |  |
| **Vegetable intake (g/day)** | |  |  |  |  |
| N | 551 | 514 | 494 | 370 | <0.001 |
| Mean (SD) | 150 (191) | 144 (171) | 142 (191) | 104 (133) |  |
|  |  |  |  |  |  |
| **Fruit intake (g/day)** |  |  |  |  |  |
| N | 551 | 514 | 494 | 370 | <0.001 |
| Mean (SD) | 118 (167) | 122 (177) | 114 (184) | 69 (126) |  |
|  |  |  |  |  |  |

SFBA, San Francisco Bay Area; LAC, Los Angeles County
